# Chemical synthesis of lipophilic methylene blue analogues which increase mitochondrial biogenesis and frataxin levels

**DOI:** 10.1016/j.dib.2018.08.156

**Published:** 2018-08-31

**Authors:** Indrajit Bandyopadhyay, Sandipan Roy Chowdhury, Nishant P. Visavadiya, Sidney M. Hecht, Omar M. Khdour

**Affiliations:** Biodesign Center for BioEnergetics, and School of Molecular Sciences, Arizona State University, Tempe, AZ 85287, USA

## Abstract

As part of an ongoing program to develop potential therapeutic agents for the treatment of the neurodegenerative disease Friedreich׳s ataxia (FRDA), we have prepared a number of lipophilic methylene blue analogues. Some of these compounds significantly increase mitochondrial biogenesis and frataxin levels in cultured Friedreich’s ataxia cells [1]. This data article describes the chemical synthesis and full physicochemical characterization of the new analogues.

**Specifications Table**

**Table:** 

Subject area	*Chemistry*
More specific subject area	*Lipophilic methylene blue analogues*
Type of data	*Synthetic schemes and methods, physicochemical characterization*
How data was acquired	*Chemical synthesis, NMR (Varian 400* *MHz), mass spectrometry (JEOL LCMate LC-MS)*
Data format	*Analyzed*
Experimental factors	*Several lipophilic methylene blue analogues were prepared by chemical synthesis, starting from 2-cyanophenothiazine*
Experimental features	*N-protected 2-cyanophenothiazine was converted to the respective aldehyde, enabling introduction of the lipophilic substituents via a Wittig reaction and of the dialkylamines at positions 3 and 7 by treatment with the amines in the presence of iodine*
Data source location	*Biodesign Center for BioEnergetics and School of Molecular Sciences, Arizona State University, Phoenix, AZ*
Data accessibility	*Data is with this article*

**Value of the data**

• The data enable the preparation of lipophilic methylene blue derivatives for evaluation in FRDA models.
• Characterization of the methylene blue analogues permitted verification of structure.
• The methods outlined should be extensible to additional new compounds of this type.

## 1 Data

**Scheme 1:**
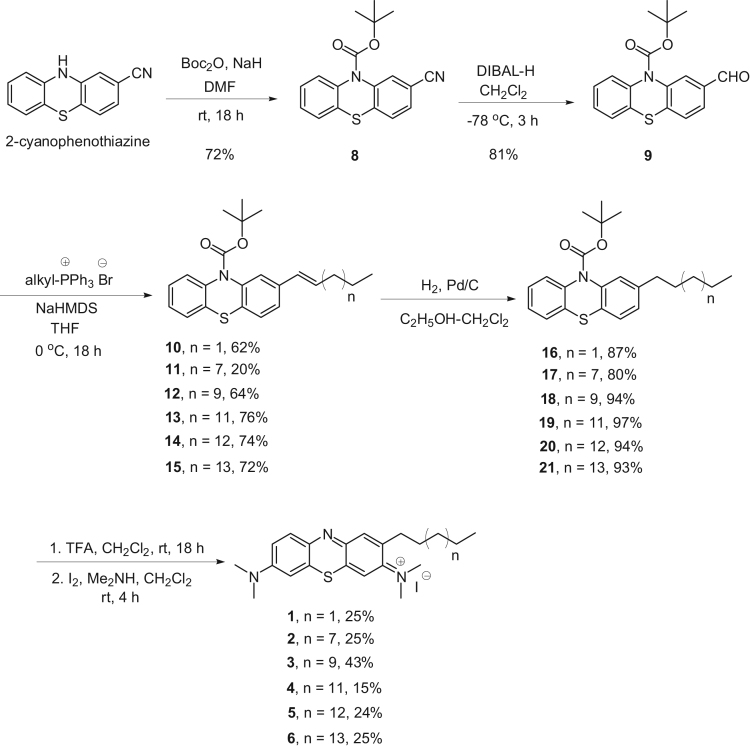
Synthetic routes employed for compounds **1**-**6**.

**Scheme 2:**
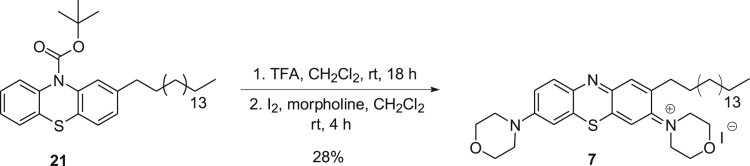
Synthetic route employed for compound **7**.

The synthetic routes employed for the preparation of lipophilic methylene blue analogues are outlined in Scheme 1, Scheme 2. Analogues **1**–**7**, each having a long alkyl substituent on a phenothiazine nucleus (Scheme 1), were prepared starting from commercially available 2-cyanophenothiazine. Initially, the N atom at position 10 was protected by treatment with NaH (60% in mineral oil) at 0 °C, followed by di-*tert*-butyl dicarbonate, affording N-Boc derivative **8** in 72% yield. Reductive hydrolysis of protected cyanophenothiazine **8** by DIBAL-H and 2N HCl afforded aldehyde **9** in 81% yield [2]. By treating **9** with each of six alkyltriphenylphosphonium bromides in the presence of 1 M NaHMDS, according to a usual protocol for Wittig reactions, the corresponding intermediate alkenes (**10–15**) were obtained as *cis-trans* mixtures. The latter were then reduced by catalytic hydrogenation over palladium-on-carbon to afford the corresponding alkanes (**16**–**21**) in good yields. In the final step, the Boc protecting group was removed with 10 equivalents of CF_3_COOH, then the intermediate was oxidized with iodine in CH_2_Cl_2_ followed by the subsequent addition of dimethylamine to afford analogues **1**–**6** (Scheme 1) [3].

Analogue 7 was obtained by treating intermediate **21** with morpholine to provide bis-morpholino derivative **7** in 28% yield (Scheme 2).

## 2 Experimental design, materials and methods

### 2.1 General experimental procedures

Reagent grade chemicals and solvents were purchased from Sigma-Aldrich Chemicals and were used without further purification. All reactions were performed under an argon atmosphere, unless otherwise specified. Thin layer chromatography (TLC) plates (precoated glass plates with silica gel 60 F254, 0.25 mm thickness) were used for analytical TLC and were visualized by UV irradiation (254 nm). Flash chromatography was carried out using Silicycle 200–400 mesh silica gel. ^1^H and ^13^C NMR spectra were obtained using a Varian 400 MHz NMR spectrometer. Chemical shifts (δ) are reported in parts per million (ppm) and are referenced to residual CHCl_3_ (δ 7.26 ppm for ^1^H NMR and δ 77.16 for ^13^C NMR) as the internal standard. Splitting patterns are designated as s, singlet; d, doublet; t, triplet; q, quartet; m, multiplet. High resolution mass spectra were obtained at the Arizona State University CLAS High Resolution Mass Spectrometry Facility.

### 2.2 Synthesis of the methylene blue analogues

#### 2.2.1 tert-Butyl 2-Cyano-10H-phenothiazine-10-carboxylate (8)

A sample of 2.00 g (8.90 mmol) of 2-cyanophenothiazine was dissolved in 25 mL of anhydrous DMF. The reaction mixture was cooled to 0 °C and 0.53 g (13.3 mmol) of 60% NaH was added. The dark reaction mixture was stirred at 0 °C for an additional 15 min and 2.33 g (10.6 mmol) of di-*tert*-butyl dicarbonate was added. The reaction mixture was stirred at room temperature for 18 h and was then diluted with 60 mL of brine. The aqueous layer was extracted with three 25-mL portions of ethyl acetate. The combined organic extract was dried over anhydrous MgSO_4_ and concentrated under diminished pressure. The crude product was purified on a silica gel column (20 × 3 cm). Elution with 9:1 hexanes-ethyl acetate gave **8** as a pale yellow solid: yield 2.10 g (72%); silica gel TLC *R*_f_ 0.26 (9:1 hexanes-ethyl acetate); ^1^H NMR (CDCl_3_) *δ* 1.49 (s, 9H), 7.16–7.20 (m, 1H), 7.28–7.33 (m, 2H), 7.40 (s, 2H), 7.49 (d, 1H, *J* = 8.4 Hz) and 7.80 (s, 1H); ^13^C NMR *δ* 28.1, 83.1, 110.2, 118.2, 126.6, 127.2, 127.3, 127.5, 128.1, 129.1, 130.3, 130.6, 137.7, 139.0, 139.1 and 151.8; mass spectrum (APCI), *m/z* 325.1017 (M+H)^+^ (C_18_H_17_N_2_O_2_S requires *m/z* 325.1011).

#### 2.2.2 tert-Butyl 2-Formyl-10H-phenothiazine-10-carboxylate (9)

To a solution of 2.30 g (7.10 mmol) of **8** in 25 mL of anhydrous CH_2_Cl_2_ was added dropwise at −78 °C 8.50 mL (8.50 mmol) of 1 M DIBAL-H in toluene. The reaction mixture was stirred at −78 °C for 3 h and was then diluted with 30 mL of brine. The aqueous layer was extracted with three 30-mL portions of CH_2_Cl_2_. The combined organic extract was dried over anhydrous MgSO_4_ and then concentrated under diminished pressure. The residue was purified on a silica gel column (20 × 3 cm). Elution with 9:1 hexane-ethyl acetate afforded **9** as a yellow solid: yield 1.88 g (81%); silica gel TLC *R*_f_ 0.17 (9:1 hexane-ethyl acetate); ^1^H NMR (CDCl_3_) *δ* 1.48 (s, 9H), 7.14–7.18 (m, 1H), 7.26–7.32 (m, 2H), 7.44 (d, 1H, *J* = 8.0 Hz), 7.52 (d, 1H, *J* = 7.4 Hz), 7.64 (d, 1H, *J* = 8.0 Hz), 7.99 (s, 1H) and 9.96 (s, 1H); ^13^C NMR *δ* 28.2, 82.9, 126.5, 126.7, 127.2, 127.3, 127.5, 127.9, 128.4, 130.6, 135.1, 138.0, 139.2, 140.3, 152.1 and 190.9; mass spectrum (ESI), *m/z* 328.1003 (M+H)^+^ (C_18_H_18_NO_3_S requires *m/z* 328.1007).

#### 2.2.3 tert-Butyl (E)-2-(Pent-1-enyl)-10H-phenothiazine-10-carboxylate (10)

A sample containing 2.30 g (5.81 mmol) of (1-butyl)triphenylphosphonium bromide was dissolved in 25 mL of anhydrous tetrahydrofuran. The cooled (−78 °C) reaction mixture was treated dropwise with 5.81 mL (5.81 mmol) of sodium bis(trimethylsilyl) amide. The reaction mixture was stirred at 0 °C for 3 h. The reaction mixture was cooled to −78 °C and 1.90 g (5.81 mmol) of **9**, dissolved in 15 mL of anhydrous tetrahydrofuran, was added. The combined reaction mixture was stirred at 0 °C for 18 h. The reaction mixture was extracted with two 30-mL portions of dichloromethane. The combined organic phase was washed with 20 mL of brine, dried over anhydrous Na_2_SO_4_ and concentrated under diminished pressure. The residue was purified on a silica gel column (20 × 3 cm). Elution with 4:1 hexane-dichloromethane afforded **10** as a yellow solid: yield 1.3 g (62%); silica gel TLC *R*_f_ 0.66 (9:1 hexane-dichloromethane); ^1^H NMR (CDCl_3_) *δ* 1.03 (t, 3H, *J* = 7.2 Hz), 1.57 (s, 11H), 2.42 (q, 2H, *J =* 7 Hz), 5.75 (m, 1H), 6.48 (d, 1H, *J =* 2 Hz), 7.15 (m, 2H), 7.32 (m, 3H), 7.58 (s, 1H) and 7.63 (d, 1H, *J =* 8 Hz); ^13^C NMR (CDCl_3_) *δ* 13.6, 22.8, 27.8, 30.3, 81.5, 125.7, 126.1, 126.3, 126.7, 127.0, 127.2, 127.7, 127.8, 129.7, 131.8, 133.2, 136.2, 138.2, 138.5 and 152.0; mass spectrum (APCI), *m/z* 368.1680 (M+H)^+^ (C_22_H_26_NO_2_S requires *m/z* 368.1684).

#### 2.2.4 tert-Butyl 2-Pentyl-10H-phenothiazine-10-carboxylate (16)

A sample containing 1.90 g (5.31 mmol) of **10** was dissolved in 20 mL of 7:3 ethanol-dichloromethane and purged with argon for 20 min. To the resulting solution was added 110 mg of 10% palladium-on-carbon. The suspension was stirred at room temperature under an atmosphere of H_2_ (40 psi) for 2 h. The reaction mixture was then filtered through a Celite pad. The filtrate was concentrated under diminished pressure. The residue was purified on a silica gel column (20 × 3 cm). Elution with 4:1 hexane-dichloromethane afforded **16** as a colorless oil: yield 1.7 g (87%); silica gel TLC *R*_f_ 0.62 (9:1 hexane-dichloromethane); ^1^H NMR (CD_3_OD) *δ* 0.76 (s, 3H), 1.16 (s, 4H), 1.32 (s, 9H), 1.45 (s, 2H), 2.41 (s, 2H), 6.77 (s, 1H), 6.94 (s, 1H), 7.05 (m, 2H), 7.14 (s, 1H), 7.23 (s, 1H) and 7.38 (s, 1H); ^13^C NMR (CD_3_OD) *δ* 14.6, 23.4, 28.5, 32.1, 32.3, 36.3, 82.8, 127.0, 127.46, 127.47, 128.0, 128.2, 128.3, 129.9, 133.5, 139.7, 139.9, 142.7 and 153.6; mass spectrum (APCI), *m/z* 370.1842 (M+H)^+^ (C_22_H_28_NO_2_S requires 370.1841).

#### 2.2.5 N-(7-(Dimethylamino)-2-pentyl-3H-phenothiazin-3-ylidene)-N-methylmethanaminium Iodide (1)

A sample containing 1.70 g (4.60 mmol) of **16** was dissolved in 20 mL of anhydrous dichloromethane. To the resulting solution was added dropwise 2.81 mL (36.8 mmol) of trifluoroacetic acid. The reaction mixture was stirred at room temperature for 18 h. The reaction was quenched with 20 mL of saturated sodium bicarbonate solution, extracted with two 30-mL portions of dichloromethane, dried over anhydrous Na_2_SO_4_ and then concentrated under diminished pressure. The crude residue was utilized for the next step without further purification.

To a solution containing 180 mg (0.66 mmol) of the crude residue in 5 mL of dichloromethane was added 543 mg (2.14 mmol) of iodine and the reaction mixture was stirred in the dark for 15 min. To the resulting solution was added dropwise 1.70 mL (3.34 mmol) of 2 M dimethylamine in THF, and the reaction mixture was stirred at room temperature for 4 h. The greenish blue mixture was purified on a silica gel column (20 × 3 cm). Elution with at 1:1 methanol-acetonitrile afforded **1** as a blue solid: yield 40.0 mg (25%); silica gel TLC *R*_f_ 0.23 (CH_3_CN); ^1^H NMR (CDCl_3_) *δ* 0.85 (t, 3H, *J =* 6.5 Hz), 1.33 (s, 4H), 1.64 (s, 2H), 2.60 (s, 3H), 2.80 (s, 2H), 3.41 (d, 9H), *J =*, 7.29 (s, 2H), 7.79 (d, 2H), and 8.67 (s, 1H); ^13^C NMR (CDCl_3_) *δ* 13.9, 22.3, 29.7, 31.5, 33.8, 34.8, 44.6, 106.9, 111.0, 119.9, 131.5, 135.2, 136.7, 137.0, 137.1, 138.4, 138.7, 153.8 and 157.8; mass spectrum (ESI), *m/z* 354.1997 (M^+^) (C_21_H_28_N_3_S requires 354.2004); ultraviolet/visible spectrum λ_max_ 665 nm (CH_2_Cl_2_), λ_max_ 665 nm (MeOH).

#### 2.2.6 tert-Butyl (E)-2-(Undec-1-enyl)-10H-phenothiazine-10-carboxylate (11)

A sample containing 792 mg (1.64 mmol) of (1-decyl)triphenylphosphonium bromide was dissolved in 12 mL of anhydrous tetrahydrofuran. The cooled (−78 °C) reaction mixture was treated dropwise with 1.64 mL (1.64 mmol) of sodium bis(trimethylsilyl) amide. The reaction mixture was stirred at 0 °C for 3 h. The reaction mixture was cooled to −78 °C and 537 mg (1.64 mmol) of **9**, dissolved in 8 mL of anhydrous tetrahydrofuran, was added. The reaction mixture was stirred at 0 °C for 18 h. The reaction mixture was extracted with two 15-mL portions of dichloromethane. The organic phase was washed with 15 mL of brine, dried over anhydrous Na_2_SO_4_ and concentrated under diminished pressure. The residue was purified on a silica gel column (20 × 1 cm). Elution with 4:1 hexane-dichloromethane afforded **11** as a yellow solid: yield 145 mg (20%); silica gel TLC *R*_f_ 0.68 (1:1 hexane-dichloromethane); ^1^H NMR (CDCl_3_) *δ* 0.93 (t, 3 H, *J* = 6.4 Hz), 1.31 (s, 13H), 1.53 (s, 10H), 2.38 (q, 2H, *J* = 7 Hz), 5.71 (m, 1H), 6.40 (d, 1H, *J* = 11.2 Hz), 7.13 (m, 2H), 7.28 (q, 2H, *J* = 7.4 Hz), 7.35 (d, 1H, *J =* 7.6 Hz), 7.49 (s, 1H) and 7.57 (d, 1H, *J =* 7.6 Hz); ^13^C NMR (CDCl_3_) *δ* 14.2, 22.8, 27.3, 28.3, 29.4, 29.7, 29.8, 29.9, 30.0, 32.0, 82.1, 126.1, 126.6, 127.3, 127.5, 127.8, 127.9, 129.9, 130.0, 130.1, 132.3, 134.0, 136.7, 138.6, 138.9 and 152.5; mass spectrum (APCI), *m/z* 452.2617 (M+H)^+^ (C_28_H_38_NO_2_S requires 452.2623).

#### 2.2.7 tert-Butyl 2-Undecyl-10H-phenothiazine-10-carboxylate (17)

A sample containing 850 mg (1.88 mmol) of **11** was dissolved in 10 mL of 7:3 ethanol-dichloromethane and purged with argon for 20 min. To the resulting solution was added 40 mg of 10% palladium-on-carbon. The suspension was stirred at room temperature under an atmosphere of H_2_ (40 psi) for 2 h. The reaction mixture was then filtered through a Celite pad. The filtrate was concentrated under diminished pressure. The residue was purified on a silica gel column (20 × 1 cm). Elution with 4:1 hexane-dichloromethane afforded **17** as a colorless oil: yield 546 mg (80%); silica gel TLC *R*_f_ 0.68 (1:1 hexane-dichloromethane); ^1^H NMR (CDCl_3_) *δ* 0.96 (t, 3H, *J =* 6.7 Hz), 1.35 (s, 16H), 1.56 (s, 9H), 1.69 (m, 2H), 2.67 (t, 2H, *J =* 7.7 Hz), 7.02 (d, 1H, *J =* 8 Hz), 7.16 (t, 1H, *J =* 7.7 Hz), 7.28 (d, 2H, *J =* 8 Hz), 7.37 (d, 1H, *J =* 8 Hz), 7.44 (s, 1H) and 7.60 (d, 1H, *J =* 7.5 Hz); ^13^C NMR (CDCl_3_) *δ* 14.1, 22.6, 28.1, 29.2, 29.3, 29.5, 29.57, 29.6, 29.7, 31.4, 31.9, 35.5, 81.7, 125.8, 126.2, 126.9, 127.0, 127.1, 127.2, 127.3, 128.7, 132.4, 138.6, 138.8, 141.6 and 152.4; mass spectrum (APCI), *m/z* 454.2772 (M+H)^+^ (C_28_H_40_NO_2_S requires 454.2780).

#### 2.2.8 N-(7-(Dimethylamino)-3H-phenothiazin-3-ylidene-2-undecyl)-N-methyl methanaminium Iodide (2)

A sample containing 820 mg (1.81 mmol) of **17** was dissolved in 12 mL of anhydrous dichloromethane. To the resulting solution was added dropwise 1.10 mL (14.5 mmol) of trifluoroacetic acid. The reaction mixture was stirred at room temperature for 18 h. The reaction was quenched with 10 mL of saturated sodium bicarbonate solution, extracted with two 20-mL portions of dichloromethane, dried over anhydrous Na_2_SO_4_ and then concentrated under diminished pressure. The crude residue was utilized for the next step without further purification.

To a solution containing 1.40 g (4.00 mmol) of the crude residue in 20 mL of dichloromethane was added 3.20 g (12.9 mmol) of iodine and the reaction mixture was stirred in the dark for 15 min. To the resulting solution was added dropwise 10.1 mL (20.3 mmol) of 2 M dimethylamine in THF and the reaction mixture was stirred at room temperature for 4 h. The greenish blue mixture was purified on a silica gel column (20 × 3 cm). Elution with methanol afforded **2** as a blue-green solid: yield 102 mg (25%); silica gel TLC *R*_f_ 0.07 (methanol); ^1^H NMR (CDCl_3_) *δ* 0.88 (t, 3H, *J =* 6.7 Hz), 1.26 (s, 18H), 1.72 (m, 2H), 2.85 (t, 2H, *J =* 7.7 Hz), 3.35 (s, 4H), 3.46 (s, 5H), 3.56 (s, 1H), 7.28 (d, 1H, *J =* 12.5 Hz), 7.37 (s, 1H), 7.39 (s, 1H), 7.87 (s, 1H) and 7.97 (d, 1H, *J =* 10 Hz); ^13^C NMR (CDCl_3_) *δ* 14.2, 22.7, 29.4, 29.5, 29.6, 29.7, 29.73, 30.2, 32.0, 34.2, 42.5, 44.8, 106.8, 111.2, 119.9, 132.0, 135.7, 137.1, 137.3, 137.4, 138.7, 138.8, 154.2 and 158.2; mass spectrum (ESI), *m/z* 438.2948 (M^+^) (C_27_H_40_N_3_S requires 438.2943); ultraviolet/visible spectrum λ_max_ 670 nm (CH_2_Cl_2_), λ_max_ 665 nm (MeOH).

#### 2.2.9 tert-Butyl (E)-2-(Tridec-1-enyl)-10H-phenothiazine-10-carboxylate (12)

A sample containing 2.65 g (5.19 mmol) of (1-dodecyl)triphenylphosphonium bromide was dissolved in 25 mL of anhydrous tetrahydrofuran. The cooled (−78 °C) reaction mixture was treated dropwise with 5.19 mL (5.19 mmol) of sodium bis(trimethylsilyl) amide. The reaction mixture was stirred at 0 °C for 3 h. The reaction mixture was cooled to −78 °C and 1.70 g (5.19 mmol) of **9**, dissolved in 15 mL of anhydrous tetrahydrofuran was added. The reaction mixture was stirred at 0 °C for 18 h. The product was extracted with two 30-mL portions of dichloromethane. The organic phase was washed with 20 mL of brine, dried over anhydrous Na_2_SO_4_ and concentrated under diminished pressure. The residue was purified on a silica gel column (20 × 3 cm). Elution with 4:1 hexane-dichloromethane afforded **12** as a yellow solid: yield 1.60 g (64%); silica gel TLC *R*_f_ 0.68 (1:1 hexane-dichloromethane); ^1^H NMR (CDCl_3_) *δ* 1.01 (t, 3H, *J =* 7 Hz), 1.38 (s, 18H), 1.58 (s, 9H), 2.45 (q, 2H, *J =* 6.8 Hz), 5.76 (m, 1H), 6.45 (t, 1H, *J =* 10 Hz), 7.14 (m, 2H), 7.31 (m, 3H) and 7.62 (q, 2H, *J =* 10.3 Hz); ^13^C NMR (CDCl_3_) *δ* 13.9, 22.5, 27.9, 28.4, 29.1, 29.2, 29.3, 29.5, 29.7, 31.7, 81.4, 125.7, 126.1, 126.2, 126.6, 127.0, 127.1, 127.2, 127.6, 129.7, 131.9, 133.4, 136.2, 138.3, 138.5 and 152.0; mass spectrum (APCI), *m/z* 480.2942 (M+H)^+^ (C_30_H_42_NO_2_S requires 480.2936).

#### 2.2.10 tert-Butyl-2-tridecyl-10H-phenothiazine-10-carboxylate (18)

A sample containing 1.60 g (3.34 mmol) of **12** was dissolved in 20 mL of 7:3 ethanol-dichloromethane and purged with argon for 20 min. To the resulting solution was added 70 mg of 10% of palladium-on-carbon. The suspension was stirred at room temperature under an atmosphere of H_2_ (40 psi) for 2 h. The reaction mixture was filtered through a Celite pad and the filtrate was then concentrated under diminished pressure. The residue was purified on a silica gel column (20 × 3 cm). Elution with 4:1 hexane-dichloromethane afforded **18** as a colorless oil: yield 1.50 g (94%); silica gel TLC *R*_f_ 0.68 (1:1 hexane-dichloromethane); ^1^H NMR (CDCl_3_) *δ* 0.97 (s, 3H), 1.35 (s, 20H), 1.56 (s, 9H), 1.69 (s, 2H), 2.67 (s, 2H), 7.02 (d, 1H, *J =* 7 Hz), 7.17 (d, 1H, *J =* 6.5 Hz), 7.28 (d, 2H, *J =* 7.5 Hz), 7.37 (d, 1H, *J =* 7 Hz), 7.45 (s, 1H) and 7.60 (d, 1H, *J =* 7 Hz); ^13^C NMR (CDCl_3_) *δ* 14.1, 22.7, 28.1, 29.31, 29.39, 29.5, 29.6, 29.7, 31.5, 31.9, 35.6, 81.7, 125.9, 126.2, 127.0, 127.1, 127.2, 127.3, 127.4, 128.8, 132.4, 138.6, 138.8, 141.6 and 152.4; mass spectrum (APCI), *m/z* 482.3092 (M+H)^+^ (C_30_H_44_NO_2_S requires 482.3093).

#### 2.2.11 N-(7-(Dimethylamino)-3H-phenothiazin-3-ylidene-2-tridecyl)-N-methylmethanaminium Iodide (3)

A sample containing 1.50 g (3.11 mmol) of **18** was dissolved in 20 mL of anhydrous dichloromethane. To the resulting solution was added dropwise 1.90 mL (24.9 mmol) of trifluoroacetic acid. The reaction mixture was stirred at room temperature for 18 h. The reaction was then quenched with 20 mL of saturated sodium bicarbonate solution, extracted with two 30-mL portions of dichloromethane, dried over anhydrous Na_2_SO_4_ and then concentrated under diminished pressure. The crude residue was utilized for the next step without further purification.

To a solution containing 1.40 g (3.67 mmol) of the crude residue in 20 mL of dichloromethane was added 3.00 g (11.7 mmol) of iodine and the reaction mixture was stirred in the dark for 15 min. To the resulting solution was added dropwise 9.20 mL (18.3 mmol) of 2 M dimethylamine in THF and the reaction mixture was stirred at room temperature for 4 h. The greenish blue mixture was purified on a silica gel column (20 × 3 cm). Elution with 1:1 ethyl acetate-methanol afforded **3** as a blue solid: yield 934 mg (43%); silica gel TLC *R*_f_ 0.03 (methanol); ^1^H NMR (CDCl_3_) *δ* 0.82 (d, 3H, *J =* 6 Hz), 1.21 (s, 20H), 1.66 (s, 2H), 2.63 (s, 4H), 2.82 (s, 2H), 3.35 (s, 4H), 3.49 (s, 4H), 7.37 (s, 2H), 7.80 (s, 1H), 7.90 (d, 1H, *J =* 1.5 Hz) and 8.63 (s, 1H); ^13^C NMR (CDCl_3_) *δ* 14.0, 22.6, 29.2, 29.3, 29.4, 29.56, 29.59, 29.61, 29.63, 30.1, 31.8, 34.0, 34.8, 42.5, 44.6, 107.0, 111.1, 119.8, 131.7, 135.4, 136.8, 137.1, 137.2, 138.5, 138.8, 154.0 and 157.9; mass spectrum (ESI), *m*/*z* 466.3250 (M^+^) (C_29_H_44_N_3_S requires 466.3256); ultraviolet/visible spectrum λ_max_ 667 nm (CH_2_Cl_2_), λ_max_ 663 nm (MeOH).

#### 2.2.12 tert-Butyl (E)-2-(Pentadec-1-enyl)-10H-phenothiazine-10-carboxylate (13)

A sample containing 2.80 g (5.19 mmol) of (1-tetradecyl)triphenylphosphonium bromide was dissolved in 25 mL of anhydrous tetrahydrofuran. The cooled (−78 °C) reaction mixture was treated dropwise with 5.19 mL (5.19 mmol) of sodium bis(trimethylsilyl) amide. The reaction mixture was stirred at 0 °C for 3 h. The mixture was cooled to −78 °C and 1.70 g (5.19 mmol) of **9**, dissolved in 15 mL of anhydrous tetrahydrofuran was added. The reaction mixture was stirred at 0 °C for 18 h. The product was extracted with two 30-mL portions of dichloromethane. The organic phase was washed with 20 mL of brine, dried over anhydrous Na_2_SO_4_ and concentrated under diminished pressure. The residue was purified on a silica gel column (20 × 3 cm). Elution with 4:1 hexane-dichloromethane afforded **13** as a yellow solid: yield 2.0 g (76%); silica gel TLC *R*_f_ 0.68 (1:1 hexane-dichloromethane); ^1^H NMR (CDCl_3_) *δ* 0.98 (t, 3H, *J =* 5 Hz), 1.36 (s, 22H), 1.56 (s, 9H), 2.42 (d, 2 H, *J =* 5 Hz), 5.74 (m, 1H), 5.44 (d, 1H, *J =* 10 Hz), 5.15 (m, 2H), 7.30 (m, 2H), 7.37 (d, 1H, *J =* 10 Hz), 7.55 (s, 1H) and 7.60 (d, 1H, *J =* 5 Hz); ^13^C NMR (CDCl_3_) *δ* 14.1 22.6, 28.1, 28.6, 29.3, 29.4, 29.5, 29.6, 29.7, 29.9, 31.9, 81.7, 125.9, 126.3, 126.4, 126.8, 126.9, 127.2, 127.3, 127.8, 129.9, 132.1, 133.6, 136.5, 138.4, 138.7 and 152.3; mass spectrum (APCI), *m/z* 508.3258 (M+H)^+^ (C_32_H_46_NO_2_S requires 508.3249).

#### 2.2.13 tert-Butyl-2-pentadecyl-10H-phenothiazine-10-carboxylate (19)

A sample containing 2.00 g (3.94 mmol) of **13** was dissolved in 22 mL of 7:3 ethanol-dichloromethane and purged with argon for 20 min. To the resulting solution was added 80 mg of 10% of palladium-on-carbon. The suspension was stirred at room temperature under an atmosphere of H_2_ (40 psi) for 2 h. The reaction mixture was filtered through a Celite pad. The filtrate was then concentrated under diminished pressure. The residue was purified on a silica gel column (20 × 3 cm). Elution with 4:1 hexane-dichloromethane afforded **19** as a colorless oil: yield 1.91 g (97%); silica gel TLC *R*_f_ 0.68 (1:1 hexane-dichloromethane); ^1^H NMR (CDCl_3_) *δ* 0.95 (t, 3H, *J =* 7.5 Hz), 1.33 (s, 24H), 1.55 (s, 9H), 1.68 (d, 2H, *J =* 5 Hz), 2.66 (t, 2H, *J =* 7.5 Hz), 7.01 (d, 1 H, *J =* 10 Hz), 7.15 (m, 1H), 7.28 (m, 2H), 7.37 (t, 1H, *J =* 5 Hz), 7.43 (s, 1H) and 7.58 (d, 1H, *J =* 10 Hz); ^13^C NMR (CDCl_3_) *δ* 14.1, 22.7, 28.2, 29.3, 29.4, 29.5, 29.6, 29.7, 29.8, 31.5, 31.9, 35.6, 81.7, 125.9, 126.3, 126.4, 127.0, 127.1, 127.2, 127.3, 128.8, 132.5, 138.6, 138.9, 141.7 and 152.4; mass spectrum (APCI), *m/z* 510.3416 (M+H)^+^ (C_32_H_48_NO_2_S requires 510.3406).

#### 2.2.14 N-(7-(Dimethylamino)-2-pentadecyl-3H-phenothiazin-3-ylidene)-N-methylmethanaminium Iodide (4)

A sample containing 1.89 g (3.71 mmol) of **19** was dissolved in 25 mL of anhydrous dichloromethane. To the resulting solution was added dropwise 2.30 mL (24.7 mmol) of trifluoroacetic acid. The reaction mixture was stirred at room temperature for 18 h. The reaction was then quenched with 20 mL of saturated sodium bicarbonate solution, extracted with two 30-mL portions of dichloromethane, dried over anhydrous Na_2_SO_4_ and then concentrated under diminished pressure. The crude residue was utilized for the next step without further purification.

To a solution containing 3.00 g (7.33 mmol) of the crude residue in 25 mL of dichloromethane was added 5.95 g (23.4 mmol) of iodine and the reaction mixture was stirred in the dark for 15 min. To the resulting solution was added dropwise 18.3 mL (36.6 mmol) of 2 M dimethylamine in THF and the reaction mixture was stirred at room temperature for 4 h. The greenish blue mixture was purified on a silica gel column (20 × 3 cm). Elution with at 1:1 ethyl acetate-methanol afforded **4** as a blue solid: yield 0.39 g (15%); silica gel TLC *R*_f_ 0.06 (methanol); ^1^H NMR (CDCl_3_) *δ* 0.87 (t, 3H, *J =* 5 Hz), 1.25 (s, 24H), 1.71 (m, 2H), 2.79 (s, 4H), 2.84 (t, 2H, *J =* 7.5 Hz), 3.34 (s, 4H), 3.48 (s, 4H), 7.27 (s, 1H), 7.38 (s, 1H), 7.41 (s, 1H), 7.88 (s, 1H) and 7.99 (d, 1 H, *J =* 10 Hz); ^13^C NMR (CDCl_3_) *δ* 14.2, 22.7, 29.4, 29.5, 29.6, 29.7, 30.2, 32.0, 34.2, 35.1, 42.6, 44.7, 106.9, 111.3, 119.8, 132.0, 135.8, 137.1, 137.3, 137.4, 138.7, 138.9, 154.2 and 158.2; mass spectrum (ESI), *m/z* 494.3576 (M^+^) (C_31_H_48_N_3_S requires 494.3563); ultraviolet/visible spectrum λ_max_ 664 nm (CH_2_Cl_2_), λ_max_ 665 nm (MeOH).

#### 2.2.15 tert-Butyl (E)-2-(Hexadec-1-enyl)-10H-phenothiazine-10-carboxylate (14)

A sample containing 1.82 g (3.30 mmol) of (1-pentadecyl)triphenylphosphonium bromide was dissolved in 20 mL of anhydrous tetrahydrofuran. The cooled (−78 °C) reaction mixture was treated dropwise with 3.30 mL (3.30 mmol) of sodium bis(trimethylsilyl) amide. The reaction mixture was stirred at 0 °C for 3 h. The mixture was cooled to −78 °C and 1.08 g (3.30 mmol) of **9**, dissolved in 15 mL of anhydrous tetrahydrofuran was added. The reaction mixture was stirred at 0 °C for 18 h. The product was extracted with two 30-mL portions of dichloromethane. The organic phase was washed with 20 mL of brine, dried over anhydrous Na_2_SO_4_ and concentrated under diminished pressure. The residue was purified on a silica gel column (20 × 3 cm). Elution with 5:1 hexane-dichloromethane afforded **14** as a colorless solid: yield 1.27 g (74%); silica gel TLC *R*_f_ 0.68 (1:1 hexane-dichloromethane); ^1^H NMR (CDCl_3_) *δ* 0.97 (t, 3H, *J =* 5 Hz), 1.35 (s, 22H), 1.53 (m, 11H), 2.41 (q, 2H, *J =* 8.3 Hz), 5.74 (m, 1H), 6.42 (t, 1H, *J =* 10 Hz), 7.15 (m, 2H), 7.30 (m, 2H), 7.38 (d, 1H, *J =* 5 Hz), 7.53 (s, 1H) and 7.60 (d, 1H, *J =* 10 Hz); ^13^C NMR (CDCl_3_) *δ* 14.1, 22.7, 28.1, 28.6, 29.3, 29.4, 29.5, 29.6, 29.71, 29.74, 29.9, 31.9, 81.8, 125.9, 126.4, 126.5, 126.8, 127.2, 127.3, 127.4, 127.7, 129.9, 132.1, 133.7, 136.5, 138.5, 138.7 and 152.3; mass spectrum (APCI), *m/z* 522.3400 (M+H)^+^ (C_33_H_48_NO_2_S requires 522.3406).

#### 2.2.16 tert-Butyl-2-hexadecyl-10H-phenothiazine-10-carboxylate (20)

A sample containing 1.27 g (2.43 mmol) of **14** was dissolved in 20 mL of 7:3 ethanol-dichloromethane and purged with argon for 20 min. To the resulting solution was added 50 mg of 10% of palladium-on-carbon. The suspension was stirred at room temperature under an atmosphere of H_2_ (40 psi) for 2 h. The reaction mixture was filtered through a Celite pad. The filtrate was then concentrated under diminished pressure. The residue was purified on a silica gel column (20 × 3 cm). Elution with 5:1 hexane-dichloromethane afforded **20** as a colorless oil: yield 1.20 g (94%); silica gel TLC *R*_f_ 0.68 (1:1 hexane-dichloromethane); ^1^H NMR (CDCl_3_) *δ* 0.95 (t, 3H, *J =* 7.5 Hz), 1.32 (s, 26H), 1.55 (s, 9H), 1.67 (m, 2H), 2.65 (t, 2H, *J =* 7.5 Hz), 7.01 (d, 1H, *J =* 10 Hz), 7.16 (t, 1H, *J =* 7.5 Hz), 7.28 (m, 2H), 7.36 (d, 1H, *J =* 5 Hz), 7.42 (s, 1H) and 7.58 (d, 1H, *J =* 10 Hz); ^13^C NMR (CDCl_3_) *δ* 14.2, 22.7, 28.2, 29.3, 29.4, 29.5, 29.6, 29.75, 29.78, 31.5, 32.0, 35.6, 81.8, 125.9, 126.3, 126.4, 127.1, 127.21, 127.24, 127.4, 128.8, 132.5, 138.7, 138.9, 141.7 and 152.5; mass spectrum (APCI), *m/z* 524.3550 (M+H)^+^ (C_33_H_50_NO_2_S requires 524.3562).

#### 2.2.17 N-(7-(Dimethylamino)-2-hexadecyl-3H-phenothiazin-3-ylidene)-N-methylmethanaminium Iodide (5)

A sample containing 1.23 g (2.35 mmol) of **20** was dissolved in 20 mL of anhydrous dichloromethane. To the resulting solution was added dropwise 1.43 mL (18.8 mmol) of trifluoroacetic acid. The reaction mixture was stirred at room temperature for 18 h. The reaction was then quenched with 20 mL of saturated sodium bicarbonate solution, extracted with two 30-mL portions of dichloromethane, dried over anhydrous Na_2_SO_4_ and then concentrated under diminished pressure. The crude residue was utilized for the next step without further purification.

To a solution containing 1.15 g (2.71 mmol) of the crude residue in 20 mL of dichloromethane was added 2.20 g (8.67 mmol) of iodine and the reaction mixture was stirred in the dark for 15 min. To the resulting solution was added dropwise 6.70 mL (13.5 mmol) of 2 M dimethylamine in THF and the reaction mixture was stirred at room temperature for 4 h. The greenish blue mixture was purified on a silica gel column (20 × 3 cm). Elution with 1:1 ethyl acetate-methanol afforded **5** as a blue solid: yield 0.36 g (24%); silica gel TLC *R*_f_ 0.06 (methanol); ^1^H NMR (CDCl_3_) *δ* 0.84 (t, 3H, *J =* 7.5 Hz), 1.22 (s, 26H), 1.67 (s, 2H), 2.67 (s, 4H), 2.83 (s, 2H), 3.36 (s, 4H), 3.52 (d, 4H), *J =* 7.37 (s, 2H), 7.83 (s, 1H), 7.93 (s, 1H) and 8.58 (s, 1H); ^13^C NMR (CDCl_3_) *δ* 14.1, 22.6, 29.3, 29.4, 29.5, 29.64, 29.68, 29.7, 30.1, 31.9, 34.1, 35.0, 42.6, 44.6, 107.0, 111.2, 119.9, 131.8, 135.5, 136.9, 137.1, 137.4, 138.6, 138.9, 154.1 and 158.1; mass spectrum (ESI), *m/z* 508.3734 (M^+^) (C_32_H_50_N_3_S requires 508.3720); ultraviolet/visible spectrum λ_max_ 667 nm (CH_2_Cl_2_), λ_max_ 664 nm (MeOH).

#### 2.2.18 tert-Butyl (E)-2-(Heptadec-1-enyl)-10H-phenothiazine-10-carboxylate (15)

A sample containing 174 mg (0.30 mmol) of (1-hexadecyl)triphenylphosphonium bromide was dissolved in 10 mL of anhydrous tetrahydrofuran. The cooled (−78 °C) reaction mixture was treated dropwise with 0.30 mL (0.30 mmol) of sodium bis(trimethylsilyl) amide. The reaction mixture was stirred at 0 °C for 3 h. The mixture was cooled to −78 °C and 100 mg (0.30 mmol) of **9**, dissolved in 7 mL of anhydrous tetrahydrofuran was added. The reaction mixture was stirred at 0 °C for 18 h. The product was extracted with two 10-mL portions of dichloromethane. The organic phase was washed with 10 mL of brine, dried over anhydrous Na_2_SO_4_ and concentrated under diminished pressure. The residue was purified on a silica gel column (20 × 1 cm). Elution with 4:1 hexane-dichloromethane afforded **15** as a yellow solid: yield 117 mg (72%); silica gel TLC *R*_f_ 0.27 (9:1 hexane-dichloromethane); ^1^H NMR (CDCl_3_) *δ* 0.93 (t, 3H, *J* = 6.6 Hz), 1.31 (s, 26H), 1.52 (s, 9H), 2.38 (q, 2H, *J* = 6.9 Hz), 5.70 (m, 1H), 6.40 (d, 1H, *J* = 11.6 Hz), 7.11 (m, 2H), 7.26 (m, 2H), 7.33 (d, 1H, *J* = 7.6 Hz), 7.50 (s, 1H) and 7.56 (d, 1H, *J* = 8 Hz); ^13^C NMR (CDCl_3_) *δ* 14.2, 22.8, 28.2, 28.7, 29.4, 29.5, 29.6, 29.70, 29.79, 29.8, 30.0, 32.0, 82.0, 126.1, 126.5, 126.6, 127.0, 127.3, 127.5, 127.6, 127.8, 130.0, 132.3, 133.9, 136.7, 138.6, 138.8 and 152.5; mass spectrum (APCI), *m/z* 536.3556 (M+H)^+^ (C_34_H_50_NO_2_S requires 536.3562).

#### 2.2.19 tert-Butyl-2-heptadecyl-10H-phenothiazine-10-carboxylate (21)

A sample containing 134 mg (0.25 mmol) of **15** was dissolved in 10 mL of 7:3 ethanol-dichloromethane and purged with argon for 20 min. To the resulting solution was added 5 mg of 10% of palladium-on-carbon. The suspension was stirred at room temperature under an atmosphere of H_2_ (40 psi) for 2 h. The reaction mixture was filtered through a Celite pad. The filtrate was then concentrated under diminished pressure. The residue was purified on a silica gel column (20 × 1 cm). Elution with 4:1 hexane-dichloromethane afforded **21** as a colorless oil: yield 134 mg (93%); silica gel TLC *R*_f_ 0.84 (9:1 hexane-ethyl acetate); ^1^H NMR (CDCl_3_) *δ* 0.88 (t, 3H, *J =* 6.8 Hz), 1.26 (s, 28H), 1.49 (s, 9H), 1.60 (m, 2H), 2.59 (t, 2H, *J =* 7.6 Hz), 6.96 (dd, 1H, *J =* 1.6 Hz), 7.12 (t, 1H, *J* = 7.4 Hz), 7.25 (m, 2H), 7.32 (dd, 1H, *J =* 1.2 Hz), 7.35 (d, 1H, *J =* 1.2Hz) and 7.52 (d, 1H, *J =* 8 Hz); ^13^C NMR (CDCl_3_) *δ* 14.1, 22.7, 28.1, 29.3, 29.4, 29.5, 29.6, 29.7, 29.78, 31.5, 31.9, 35.6, 81.7, 125.8, 126.2, 126.3, 127.0, 127.1, 127.19, 127.3, 128.8, 132.4, 138.6, 138.8, 141.6 and 152.4; mass spectrum (APCI), *m/z* 538.3723 (M+H)^+^ (C_34_H_52_NO_2_S requires 538.3719).

#### 2.2.20 N-(7-(Dimethylamino)-2-heptadecyl-3H-phenothiazin-3-ylidene)-N-methylmethanaminium Iodide (6)

To a solution of 0.23 g (0.43 mmol) of **21** in 8 mL of anhydrous CH_2_Cl_2_ was added dropwise 0.26 mL (3.44 mmol) of trifluoroacetic acid. The reaction mixture was stirred at room temperature for 12 h under an argon atmosphere and quenched with 50 mL of saturated NaHCO_3_ solution. The aqueous layer was extracted with three 30-mL portions of CH_2_Cl_2_. The combined organic layer was dried over anhydrous MgSO_4_ and then concentrated under diminished pressure. The crude residue was utilized in the next step without further purification.

To a solution of 45.0 mg of the crude residue in 5 mL of CH_2_Cl_2_ was added 81.0 mg (0.32 mmol) of iodine followed by 0.25 mL (0.50 mmol) of 2 M dimethylamine in THF. The reaction mixture was stirred at room temperature under an argon atmosphere for 12 h. The greenish blue mixture was purified on a silica gel column (10 × 2 cm). Elution with ethyl acetate afforded **6** as a green solid: yield 16 mg (25%); silica gel TLC *R*_f_ 0.40 (ethyl acetate); ^1^H NMR (CDCl_3_) *δ* 0.86 (t, 3H, *J* = 6.6 Hz), 1.15–1.37 (m, 28H), 1.69–1.72 (m, 2H), 2.81–2.85 (m, 2H), 3.33 (s, 6H), 3.46 (s, 6H), 7.36–7.38 (m, 1H), 7.41 (s, 1H), 7.76 (m, 1H), 7.89 (s, 1H) and 7.99 (d, 1H, *J* = 7.6 Hz); ^13^C NMR (CDCl_3_) *δ* 14.2, 22.7, 29.4, 29.6, 29.70, 29.74, 29.78, 29.79, 30.2, 32.0, 34.1, 34.8, 42.6, 44.7, 107.4, 111.5, 119.9, 132.1, 135.92, 135.94, 137.1, 137.4, 138.5, 138.9, 154.1 and 158.1; mass spectrum (APCI), *m/z* 522.3882 (M^+^) (C_33_H_52_N_3_S requires *m/z* 522.3882); ultraviolet/visible spectrum λ_max_ 670 nm (CH_2_Cl_2_), λ_max_ 665 nm (MeOH).

#### 2.2.21 4-(2-Heptadecyl-7-morpholino-3H-phenothiazin-3-ylidene)morpholin-4-ium Iodide (7)

To a solution of 0.23 g (0.43 mmol) of **21** in 8 mL of anhydrous CH_2_Cl_2_ was added dropwise 0.26 mL (3.44 mmol) of trifluoroacetic acid. The reaction mixture was stirred at room temperature for 12 h under an argon atmosphere and then neutralized with 50 mL of saturated NaHCO_3_ solution. The aqueous layer was extracted with three 30-mL portions of CH_2_Cl_2_. The combined organic layer was dried over anhydrous MgSO_4_ and concentrated under diminished pressure. The crude product was utilized in the next step without further purification.

To a solution of 82.0 mg of the crude product in 8 mL of CH_2_Cl_2_ was added 144 mg (0.57 mmol) of iodine followed by 61.0 µL (0.72 mmol) of morpholine. The reaction mixture was stirred at room temperature under an argon atmosphere for 3 h. The greenish blue mixture was purified on a silica gel column (20 × 1 cm). Elution with at 1:1 ethyl acetate-methanol afforded **7** as a dark green solid: yield 38 mg (28%); silica gel TLC *R*_f_ 0.13 (1:1 ethyl acetate-methanol); ^1^H NMR (CDCl_3_) *δ* 0.88 (t, 3H, *J* = 6.8 Hz), 1.24–1.40 (m, 30H), 1.76–1.80 (m, 7H), 2.76 (t, 2H, *J* = 7.8 Hz), 3.43–3.45 (m, 3H), 3.92–3.98 (m, 6H), 7.56 (s, 1H), 7.61–7.64 (m, 1H), 7.69 (d, 1H, *J* = 2.0 Hz), 8.06 (s, 1H) and 8.13 (d, 1H, *J =* 9.6 Hz); ^13^C NMR (CDCl_3_) *δ* 14.1, 22.7, 29.3, 29.4, 29.5, 29.6, 29.7, 30.1, 30.9, 31.9, 32.1, 48.9, 52.3, 66.6, 107.7, 113.7, 121.2, 130.9, 137.6, 137.9, 139.7, 139.8, 140.1, 153.9 and 157.9; mass spectrum (ESI), *m/z* 606.4113 (M^+^) (C_37_H_56_N_3_O_2_S requires *m/z* 606.4093); ultraviolet/visible spectrum λ_max_ 670 nm (CH_2_Cl_2_), λ_max_ 665 nm (MeOH).
